# Dietary Recommendations from the Moroccan Society for Rheumatology (SMR) for Patients with Chronic Inflammatory Rheumatic Diseases

**DOI:** 10.31138/mjr.030624.dra

**Published:** 2025-03-31

**Authors:** Hind El-Kasmi, Samira Rostom, Salma Zemrani, Bouchra Amine, Latifa Tahiri, Nessrine Akasbi, Kawtar Nassar, Soumiya Mehdioui, Sara Wakrim, Racha Lahlou, Nada Bensaoud, Rachina Bahiri

**Affiliations:** 1 Department of Rheumatology A, El Ayachi Hospital, Ibn Sina University Hospital, Rabat, Morocco;; 2 Department of Rheumatology B, El Ayachi Hospital, Ibn Sina University Hospital, Rabat, Morocco;; 3 Department of Rheumatology, Hassan II University Hospital, Fes, Morocco;; 4 Department of Rheumatology, Ibn Rochd University Hospital, Casablanca, Morocco;; 5 Private Practice, Agdal, Rabat, Morocco;; 6 Private Practice, Kenitra, Morocco

**Keywords:** nutrition, recommendations, chronic inflammatory rheumatic diseases, dietary recommendations, rheumatoid arthritis, spondyloarthritis, psoriatic arthritis, diet

## Abstract

This work was conducted under the auspices of the Moroccan Society of Rheumatology (SMR) with the aim of developing best practice medical guidelines for the dietary management of patients with Chronic Inflammatory Rheumatic Diseases (IRDs). A working group composed of rheumatology experts and two nutritionists was formed. This group relied on a synthesis of the literature and expert opinions. These guidelines were then validated by a group of rheumatology experts. The methodology followed the procedure proposed by the European League Against Rheumatism (EULAR). Five general principles and eleven recommendations were established. These principles emphasise that diet should complement, rather than replace, pharmacological treatment and should be integrated into the overall management of patients with IRDs. Additionally, it should be associated with appropriate physical activity and take cultural and socio-economic context into account. These recommendations highlight the importance of weight loss for overweight or obese patients and advocate for the Mediterranean diet as well as a diet rich in omega-3. However, exclusion diets such as gluten-free, vegetarian, and dairy-free diets, as well as supplementation with probiotics or spices, are currently not recommended for patients with IRDs. Supplementation with vitamins or trace elements is not systematically recommended, and the data concerning Ramadan fasting or intermittent fasting are limited or contradictory. Furthermore, two specific recommendations for the Moroccan diet were proposed. These recommendations standardise dietary management in IRDs, making it accessible for practitioners and patients.

## INTRODUCTION

Rheumatic diseases encompass a wide range of immune-mediated disorders affecting the musculoskeletal system, leading to pain, disability, and a significant decline in health-related quality of life.^1^ Among them are chronic Inflammatory Rheumatic Diseases (IRDs), which include many conditions. However, in these recommendations, we will specifically focus on Rheumatoid Arthritis (RA), Spondyloarthritis (SpA), and Psoriatic Arthritis (PsA). Despite therapeutic advancements, such as the optimal use of Disease-Modifying Anti-Rheumatic Drugs (DMARDs) with the “treat-to-target” approach, a considerable number of IRDs patients are unable to achieve remission through pharmacological interventions alone.^2^

Moroccan and French recent recommendations highlight the crucial role of non-pharmacological measures in the overall management of patients with IRDs.^3–5^

Scientific interest has been growing in understanding the role of nutrition in IRDs, suggesting its substantial impact on the pathogenesis and prognosis of these diseases. However, the current understanding of the potential associations between gut microbiota and rheumatic diseases remains incomplete.^6,7^ Postulated biological mechanisms link gut dysbiosis to systemic inflammation. The gut epithelial cells form a dynamic physical barrier to control antigen passage through paracellular pathways. The breaching of this tight control causes what we call dysbiosis, which has been observed in various rheumatic diseases, including RA and SpA.^6^

Given the close relationship between diet and IRDs, numerous studies have explored their associations. The first international dietary recommendations for patients with IRDs, consisting of eight general principles and nine recommendations were published by the French Society for Rheumatology (SFR) in 2022.^8^

In our specific context, the Moroccan population is increasingly concerned about nutrition. A total of 22.8% have discussed dietary considerations with their rheumatologists, while 89.1% have expressed interest in such discussions.^9^

Several diets (Mediterranean, vegetarian, and gluten-free) have been studied. However, most studies lack strong levels of evidence, underscoring the need for these recommendations. Various studies were analysed and reviewed to deduce practical recommendations that could aid practitioners in routine practice. Given the growing interest in nutrition in recent years, primarily influenced by social media, these recommendations may be updated as more literature becomes available.

The aim of our recommendations was to develop practical and accessible guidelines tailored to the national context. These recommendations serve as a valuable tool for health professionals, including rheumatologists, physician nutrition specialists, dietitians and global practitioners.

## METHODS

These recommendations were developed by adhering to the updated procedures proposed by the European League Against Rheumatism (EULAR).^10^

### Steering Committee

The convener (SR) initiated the formation of a steering committee to establish the initial nutrition recommendations for patients with IRDs under the auspices of the Moroccan Society of Rheumatology. The working group, consisting of 10 rheumatology experts from both private and public sectors, along with 2 registered dietitians, conducted a systematic literature review led by three fellows (H.E, S.R, and S.Z). This systematic review followed the PRISMA (Preferred Reporting Items for Systematic Reviews and Meta-Analyses) recommendations,^11^ utilising the Medline database (via PubMed and Google Scholar). This review aimed to answer pre-validated questions by the working group regarding the effectiveness of diet/supplementation on the activity of chronic inflammatory rheumatisms (RA, SpA, and PsA) and on comorbidities (cardiovascular and metabolic).

The evidence for each recommendation was initially rated by the Oxford Levels of Evidence based on the type of research question,^12^ and the strength of each recommendation was defined, as presented in **Table 1**.

**Table 1.** Dietary Recommendations from the Moroccan Society for Rheumatology (SMR) for Patients with Chronic Inflammatory Rheumatic Diseases.

	**Grade**	**Agreement of working group**	**Agreement of review board**
**Overarching principles**			
A. Dietary advice should not be a substitute for pharmacological therapy of chronic inflammatory rheumatic diseases.	NA	9.9 ± 0.2	9.7 ± 0.3
B. Dietary advice provided to patients with chronic inflammatory rheumatic diseases should be based on scientific literature data.	NA	9.7 ± 0.4	9.5 ± 0.7
C. Nutrition should be integrated into the overall management of patients with chronic inflammatory rheumatic diseases. Collaboration between treating rheumatologists and dietitians should be encouraged whenever possible.	NA	9.5 ± 0.4	9.3 ± 1.0
D. Dietary advice should always be associated with adapted physical activity.	NA	9.7 ± 0.3	9.7 ± 0.5
D. Dietary advice should consider the cultural and socio-economic context of patients.	NA	9.7 ± 0.7	9.6 ± 1.3
**RECOMMENDATIONS**			
**1. Weight loss:** Weight loss should be suggested to obese or overweight patients due to its beneficial effects on chronic inflammatory rheumatic disease activity and cardiometabolic comorbidities.	B	9 ± 0.4	9.7 ± 0.7
**2. Mediterranean diet:** The Mediterranean-type diet could be suggested for patients with rheumatoid arthritis because of its beneficial effects on disease activity and cardiometabolic comorbidities. However, there is insufficient scientific literature data to support its recommendation for managing activity in spondyloarthritis and psoriatic arthritis.	B	9.1 ± 0.8	9.1 ± 1.2
**3. Exclusion diets:** Exclusion diets such as gluten-free (except in cases of associated celiac disease), vegetarian, and dairy-free diets should not be recommended for managing the activity of chronic inflammatory rheumatic diseases.	B	9.5 ± 0.7	9.4 ± 1.0
**4. Vitamins and trace elements supplementation:** Supplementation with vitamins (D, E, K, and folic acid) or trace elements (selenium, coenzyme Q10, and zinc) is not systematically recommended for patients with chronic inflammatory rheumatic diseases. A balanced diet, rich in vitamins and trace elements, should be preferred.	B	9.5 ± 0.4	9.7 ± 0.9
**5. Omega-3:** A diet rich in omega-3 is recommended for patients with chronic inflammatory rheumatic diseases. For patients considering dietary supplementation, essential polyunsaturated fatty acids, especially omega-3 at a dose greater than 2g per day, could be proposed to manage symptoms in rheumatoid arthritis.	A	8.7 ± 1.3	8.4 ± 1.5
**6. Probiotics:** Probiotics are not currently recommended for managing the disease activity in patients with chronic inflammatory rheumatic diseases (insufficient or contradictory scientific literature data).	B	9.4 ± 0.5	9.4 ± 0.8
**7. Spices:** Spices with anti-inflammatory properties (saffron, cinnamon, garlic, ginger, and curcuma) could have a beneficial effect in patients with rheumatoid arthritis, provided that appropriate doses are used and contraindications are respected, especially for pregnant and breastfeeding women, the elderly, patients with comorbidities, and those on multiple medications. However, there is not enough data to recommend them for routine practice. Insufficient scientific data for spondyloarthritis and psoriatic arthritis.	C	9.3 ± 1.0	9.2 ± 1.3
**8. Intermittent and Ramadan fasting:** Scientific data on intermittent fasting or during Ramadan is insufficient or contradictory. However, it is recommended to associate fasting with a healthy and balanced diet.	D	9.2 ± 0.9	9.5 ± 1.2
**9. Beverages:** -For patients with chronic inflammatory rheumatic diseases, it is recommended to limit the consumption of Sugar-Sweetened Beverage because of their high sugar content, which can lead to weight gain and increase the risk of cardio-metabolic diseases. -There are insufficient data in the literature to draw conclusions about caffeine and tea. -Overall, it is advisable to maintain a good hydration level, giving priority to water.	C	9.3 ± 1.0	9.6 ± 0.8
**10. Specific recommendation for Moroccan nutrition:** -For patients with chronic inflammatory rheumatic diseases, it is advisable to replace white bread with whole grain bread. -The consumption of olive oil can be recommended to patients (with 1 to 2 tablespoons per day). -Red meat consumption should be balanced and adapted according to the patient’s condition and associated comorbidities.	D	9 ± 0.3	8.8 ± 0.7
**11. Specific recommendation for Moroccan nutrition:** Scientific data on flaxseed and nigella, widely used in traditional Moroccan medicine, is insufficient or contradictory to recommend them to patients with chronic inflammatory rheumatic diseases.	D	9 ± 0.6	8.5 ± 1.2

The recommendation grade reflects the level of evidence (Mean ± SD), NA: not applicable. Agreement score, a scale between 0 (no agreement) to 10 (complete agreement).

Pre-vote meetings aimed to present the literature results, discuss different elements, and propose formulations for the items of recommendations.

### Vote processing

The first round of voting occurred after the initial meeting. For an item to be retained, more than 75% of participants had to be in favour of it. If necessary, recommendations were reformulated.^8,10^ An exemplar of proposed recommendations with analysed literature references was then sent via email. Subsequently, a second round of voting took place using Google Forms, where the working group assigned a score from 0 to 10 for each recommendation. A score of 0 indicated complete disagreement, and 10 indicated complete agreement, accompanied by an explanatory comment if the score was below 5. Recommendations with a score of under 5 were reformulated, and a third vote was conducted using Google Forms. The results of this final vote are presented in **Table 1.**

### Review process

The recommendations underwent a review by a board consisting of 10 rheumatology experts from both public and private sectors. Board members cast votes on a scale, from 0 (complete disagreement) to 10 (complete agreement). Their feedback and comments were considered during the development of the article. The voting results are presented in **Table 1.**

## RESULTS

This process led to the formulation of five principles and eleven recommendations. The strength of each recommendation, along with the agreements reached by both the working group and review board, is presented in **Table 1.**

## OVERARCHING PRINCIPLES

### Principle A: Dietary advice should not be a substitute for pharmacological therapy of chronic inflammatory rheumatic diseases.

The initial general principle highlights the importance of understanding that the nutritional aspect serves as a supplementary component of medical care. None of these dietary models or supplements has demonstrated any effect on structural impact. This is particularly crucial in managing chronic inflammatory rheumatism, where the window of opportunity is crucial, and under no circumstances is it acceptable to substitute or delay medical treatment.

### Principle B: Dietary advice provided to patients with chronic inflammatory rheumatic diseases should be based on scientific literature data.

There are many misconceptions about nutrition in our society and patients often seek information from less reliable sources such as social media. The aim of these recommendations is to provide rheumatologists with a tool to give their patients nutritional advice based on sound scientific evidence.

### Principle C: Nutrition should be integrated into the overall management of patients with chronic inflammatory rheumatic diseases. Collaboration between treating rheumatologists and dietitians should be encouraged whenever possible.

This principle highlights two key elements. Firstly, it emphasises the significance of integrating nutrition into overall management, including various aspects such as psychological well-being and adapted physical activity, which plays a pivotal role in patient care.

Secondly, the working group advocates for the promotion of collaboration between rheumatologists and dietitians, to the extent possible, especially within tertiary care structures. The introduction of nutrition into patient care encourages active patient participation in their management and promotes a transition to a healthier lifestyle.

### Principle D: Dietary advice should always be associated with adapted physical activity.

In recent years, there has been increasing interest in incorporating physical activity as an adjunctive therapy for IRDs. It’s seen as a complementary aspect to dietary measures, both of which are considered essential.

### Principle E: Dietary advice should consider the cultural and socio-economic context of patients.

During the realisation of these recommendations, the working group stressed that the recommendations could be applied regardless of cultural and socio-economic backgrounds. While two recommendations specifically address the diet and habits of Moroccan society, it’s important to note that the working group favoured a focus on natural nutrition over supplementation, making it adaptable to various socio-economic contexts. The aim was to ensure that the recommendations are practical and accessible to a wide range of populations, recognizing the diversity of available food resources.

## RECOMMENDATIONS

### Recommendation 1: Weight loss should be suggested to obese or overweight patients due to its beneficial effects on chronic inflammatory rheumatic disease activity and cardiometabolic comorbidities.

Morocco has a higher prevalence of obesity compared to the regional average, with rates of 20.8% for women and 9.2% for men.^13^ This recommendation is based on data from several studies, the first Randomised Controlled Trial (RCT) involved 40 RA patients with a *Body mass index* (BMI) ≥ 30 and a Disease Activity Score (DAS28) ≥ 3.2, equally divided between those on a low-calorie diet and those in the control group. ^14^ The study was conducted over a 12-week follow-up period, at the end of which patients on the diet had lost an average of 9.5 kg/patient, compared with 0.5 kg in the control group (p < 0.001) and a significant decrease in DAS28 was observed in the diet group. However, there was no significant difference in ultrasound measurements. The second clinical trial involving 138 obese or overweight patients with PsA, who were divided into two groups: one on a calorie-restricted diet and the other with no dietary restrictions. The results showed that patients who lost more than 5% of their body weight, regardless of group, were four times more likely to achieve minimal disease activity (MDA) than those who did not (odds ratio = 4.20 [1.82–9.66], p < 0.001).^15^ Observational studies have also demonstrated the beneficial effect of weight loss on RA and PsA activity.^16–18^ In addition, a recent systematic review of patients with SpA and PsA highlighted that weight loss, but not the adoption of a low-calorie diet, was associated with the achievement of MDA in PsA.^19^

However, it is important to note that certain situations, such as sarcopenia or malnutrition, and certain conditions, such as the elderly and pregnant women, are not addressed in these recommendations.

### Recommendation 2: The Mediterranean-type diet could be suggested for patients with rheumatoid arthritis because of its beneficial effects on disease activity and cardiometabolic comorbidities. However, there is insufficient scientific literature data to support its recommendation for managing activity in spondyloarthritis and psoriatic arthritis.

The Mediterranean diet (MD) is a dietary model characterised by a daily consumption of fish, olive oil, fruits, vegetables, whole grains, legumes, and nuts. We present in **Table 2** a pyramid model of MD, established by the Mediterranean Diet Foundation (MDF).^20,21^ It is recognised for its anti-inflammatory properties and is considered one of the most recommended diets for cardiovascular health.^22^

**Table 2.** Pyramid model of the Mediterranean diet.

**Foods**	**Mediterranean Diet Foundation (2011)**
Vegetables	≥ servings every meal
Fruits	1–2 servings every meal
Breads and cereals	1–2 servings every meal
Legumes	≥servings weekly
Olive oil	Every meal
Nuts	1–2 servings daily
Fish/Seafood	≥2 servings weekly
Eggs	2–4 servings weekly
Poultry	2 servings weekly
Dairy foods	2 servings daily
Red meat	<2 servings/week
Sweets	<2 servings/week
Water	Daily intake of 1,5–2 litres

This Pyramid model was established by the Mediterranean Diet Foundation (MDF) in 2011.^20^

Serving sizes specified as: 25g bread, 100g potato, 50–60g cooked pasta, 100g vegetables, 80g apple, 60g banana, 100g orange, 200g melon, 30g grapes, 1 cup milk or yoghurt, 1 egg, 60g meat, 100g cooked dry beans.^21^

Over the years, several clinical trials have assessed the effects of the MD, whether alone or in combination with other interventions, in patients with IRDs, especially RA. Three older clinical trials highlighted an improvement in disease activity (DAS 28) and functional impact (Health Assessment Questionnaire [HAQ]) in RA patients following a MD.^23–25^

Among more recent clinical trials, a study showed a significant reduction in DAS28 during intervention periods compared to control periods when the MD was combined with probiotics.^26^Another study compared three groups: MD, “low-fat” diet, and a control group, demonstrating a decrease in the DAS 28 score in the MD group compared to the “low-fat” group, independent of weight loss.^27^Two clinical trials explored the MD in combination with sustained physical activity. In this regard, one study did not show a significant improvement in the quality of life in the MD group, unlike the MD group combined with sustained physical activity.^28^ Furthermore, another RCT revealed an improvement in DAS 28 in the MD group combined with sustained physical activity compared to the control group.^29^

Additional observational studies were conducted, including a prospective study on patients with SpA, demonstrating an improvement of over 20% in Anky-losing Spondylitis Disease Activity Score (ASDAS) in the MD group, based on the PREDIMED questionnaire.^30^ Two other studies involving patients with RA revealed an improvement in disease activity (DAS 28) by assessing adherence to the MD using the MedDiet Questionnaire,^31,32^ as well as functional impact with the Rheumatoid Arthritis Impact of Disease questionnaire (RAID) and the HAQ.^32^

Regarding cardiovascular health, numerous high-quality studies have established the superiority of the MD in preventing cardiovascular events.^22,34^ However, it is essential to note that the specific content of the MD may vary from one study to another depending on the sociocultural context, while maintaining its fundamental principles.

### Recommendation 3: Exclusion diets such as gluten-free (except in cases of associated coeliac disease), vegetarian, and dairy-free diets should not be recommended for managing the activity of chronic inflammatory rheumatic diseases.

For the gluten-free diet, no randomised study has specifically examined the isolated impact of such a diet on patients with chronic IRDs. Previous trials have assessed the effects of a gluten-free diet when combined with a vegetarian diet in patients with RA.

Hafstrom et al conducted a RCT involving 66 RA patients, with 38 assigned to a vegan gluten-free diet and 28 to a well-balanced non-vegan diet.^35^ The follow-up period was 9 months or more, and the American College of Rheumatology (ACR) 20 improvement criteria were met by 40.5% of those on the vegan gluten-free diet compared to only 4% on the non-vegan diet. Another RCT showed an improvement in disease activity assessed by DAS 28, but the analysis was limited to patients who completed the study, restricting the generalisability of conclusions. Both studies noted a significant weight loss in the interventional groups, potentially attributable to this observed effect.^36^

It is important to note that these studies are older, and patients were not optimally treated, resulting in outcomes that fall far short of current treatment targets. A recent literature review aimed to assess the gluten-free diet in RA patients. The review included 16 articles, concluding that there is insufficient scientific evidence to support the recommendation of a gluten-free diet for patients with RA.^37^

Regarding the vegan diet, its beneficial effect could be explained by antioxidants components and dietary fibers that contribute to increasing anti-inflammatory short chain fatty acids, and reducing pro-inflammatory cytokines and positively modifying the composition of the gut microbiome.^38^ In practical, studies have evaluated vegan diet combined to fasting and gluten free diet,^39^ Lactobacillus,^40^ or fish oil.^41^

The first study was a randomised, single-blind controlled trial lasting 7–10 days. During this period, participants underwent a subtotal fast followed by a gluten-free vegan diet for 3.5 months, succeeded by a lactovegetarian diet for 9 months, in comparison to an ordinary diet. The results demonstrated significant improvements in both clinical and biological measures after the fasting phase, and these improvements persisted through dietary interventions. In contrast, the control group experienced a noteworthy improvement only in pain scores.^39^ It is crucial to note that a per-protocol analysis was conducted, excluding a substantial number of patients who discontinued the study, there- by complicating the interpretation of the results.

The two additional RCTs were of short duration. The first investigated an uncooked vegan diet rich in lacto-bacilli compared to a control group over 2–3 months, revealing no significant changes in parameters related to rheumatic disease activity.^40^ And, the second lasted 3 months and explored a vegan diet in combination with omega-3 supplementation, the groups not receiving omega-3 supplementation showed improvement only in pain levels on the Visual Analog Scale (VAS) within the vegetarian diet compared to the Western diet. However, no effect was observed on the number of painful or swollen joints, or on the HAQ.^41^

Another single-blind dietary intervention study assessed a 4-week period of a very low-calorie vegan diet. Significant weight reduction was observed, along with improvements in clinical parameters, except for morning stiffness (p > 0.05). Notably, there were no significant changes in C-reactive protein (CRP) and erythrocyte sedimentation rate (ESR).^42^

It is crucial to reiterate that all the studies discussed were conducted in the past, during a period when the treatment for RA may not have been optimal.

Data on dairy products may be conflicting. A systematic review of dietary interventions in overweight or obese individuals found that dairy consumption had no adverse effects on inflammatory biomarkers.^43^ On the other hand, red meat, eggs and dairy products are important sources of trimethylamine N-oxide (TMAO), a pro-inflammatory metabolite resulting from the metabolism of choline and carnitine.^44^ However, there is a lack of data to make practical recommendations regarding the elimination of dairy products.

A RCT of 40 patients with long-standing RA compared the effects of a 3-month diet excluding meat, gluten, lactose and all dairy products with a balanced control diet that included these foods. Both diets improved quality of life compared to baseline, with patients experiencing significant reductions in body weight and body mass index, as well as reductions in waist and hip circumference.^45^

Regarding comorbidities, a meta-analysis of RCTs showed that fermented milk consumption was associated with a reduced risk of stroke, ischaemic heart disease and cardiovascular mortality. In addition, yoghurt consumption was associated with a reduced risk of developing type 2 diabetes and metabolic syndrome.^46^

### Recommendation 4: Supplementation with vitamins (D, E, K, and folic acid) or trace elements (selenium, coenzyme Q10, and zinc) is not systematically recommended for patients with chronic inflammatory rheumatic diseases. A balanced diet, rich in vitamins and trace elements, should be preferred.

#### Vitamins supplementation

##### Vitamin D

Three meta-analyses were identified,^47 –49^ with two examining all studies including chronic inflammatory rheumatic diseases, and one on studies related to RA. The first meta-analysis included studies on RA, systemic lupus erythematosus, and systemic sclerosis. Among RA patients, a meta-analysis of three RCTs indicated no significant reduction in VAS pain or DAS 28.^47^ The second meta-analysis assessed vitamin supplementation in patients with IRDs (RA, SpA, and PsA), comprising eight studies on vitamin D supplementation. The analysis of DAS 28 in three studies, showing no heterogeneity (I2 = 0%), indicated no statistically significant improvement. Regarding VAS pain, evaluation across five studies, with high heterogeneity (I2 = 88%), demonstrated no statistically significant improvement.^48^

In another meta-analysis focusing on RA patients, six studies were included, revealing a significant association between vitamin D supplementation and DAS 28 improvement. Notably, there was high heterogeneity among the five studies (I2 = 87%). Although the VAS pain in the vitamin D supplement group was lower than that in the control group, but the difference was not statistically significant.^49^

There is insufficient evidence to recommend systematic supplementation for patients with IRDs. However, vitamin D supplementation is always recommended in cases of deficiency.

##### Vitamin E

In the initial meta-analysis,^48^ the efficacy of vitamin E supplementation in VAS pain was assessed through two studies, no significant difference was found, with high heterogeneity in the studies (I2 = 80%). A more recent meta-analysis involved nine studies encompassing a total of 39,845 RA patients,^50^ the only significant findings were a reduction in sensitive joints in the Vitamin E supplementation group. Data is limited in providing a conclusive statement regarding the impact of vitamin E on IRDs.

##### Vitamin K

Only two RCTs have investigated the effects of vitamin K in RA.^51,52^ The first trial, involving 58 RA patients, explored the effect of vitamin K1 versus placebo on lipid-related markers. No significant difference was observed between the groups, and furthermore, no difference was found in DAS28.^51^The second trial included 64 RA patients and focused on the effect of vitamin K1 on the biomarker of joint destruction (serum levels of matrix metalloproteinase-3 [MMP-3]). There was no significant change observed from baseline. Although the DAS 28 appeared to show a reduction in the vitamin K group compared to baseline, this reduction did not reach statistical significance when compared to the placebo group.^52^

##### Folic Acid

Two RCTs assessed folic acid supplementation in association with methotrexate. The first RCT involved 79 RA patients, revealing significantly lower toxicity in the folic acid supplementation group compared to the placebo.^53^The second trial, which included 40 RA patients, showed that a reduction in folic acid dose was not associated with a change in methotrexate-related adverse effects.^54^ In terms of disease activity, no significant improvement was observed in DAS28 in either of the two RCTs.

#### Trace elements supplementation

##### Selenium

Selenium is an essential trace element with antioxidant functions, playing a crucial role as a component of GPx (Glutathione peroxidase). It also demonstrates anti-inflammatory properties by inhibiting the NF-κB cascade (nuclear factor-kappa B) and reducing the production of inflammatory mediators.^55,56^ Due to these properties, there is a hypothesis that selenium may have an anti-inflammatory effect in chronic inflammatory disorders such as RA.^56^

Four RCTs investigated the impact of selenium supplementation on RA activity.^57–60^ Among them, three did not identify a significant difference between the selenium group and the placebo group.^57–59^ In one RCT with a 3-month follow-up of selenium intake (200 μg/day enriched yeast), a significant difference was observed only in two items of a quality-of-life questionnaire.^60^

##### Others

Concerning zinc supplementation, two trials with small sample sizes did not find significant joint effects in RA.^61,62^ Coenzyme Q10 supplementation was also assessed in RA patients as a potential antioxidant and anti-inflammatory substance.^63^ Two RCTs were found, revealing improvements in DAS 28 and inflammatory biomarkers (ESR),^64^ along with a decrease in cytokine levels (Tumour Necrosis Factors [TNF]) and malondialdehyde.^65^ Regarding potassium supplementation, an RCT investigated potassium supplementation in female hypokalaemic patients with active RA. The study reported substantial effects, including pain reduction, disease activity improvement, and reductions in tender and swollen joint counts.^66^

In PsA, an RCT explored the supplementation of selenium, co-enzyme Q10, and vitamin E for patients with psoriasis and joint involvement, including radiographic erosion. The study reported a significant impact on disease severity but no effect on psoriasis severity.^67^

In summary, the authors concluded that currently, there is insufficient data to systematically recommend vitamin or trace element supplementation in patients with IRDs. However, they suggest that future randomised controlled trials with larger sample sizes are desirable.

### Recommendation 5: A diet rich in omega-3 is recommended for patients with chronic inflammatory rheumatic diseases. For patients considering dietary supplementation, essential polyunsaturated fatty acids, especially omega-3 at a dose greater than 2g per day, could be proposed to manage symptoms in rheumatoid arthritis.

Polyunsaturated fatty acids (PUFAs) comprise two series: omega-6 (mainly found in vegetable oils) and omega-3 (mainly found in fish and marine algae oils) fatty acids. Omega-3 fatty acids, such as alpha-linolenic acid (ALA), eicosapentaenoic acid (EPA), and docosahexaenoic acid (DHA), are essential components of a healthy diet. The dietary sources of omega-3 essential PUFAs are detailed in **Table 3** according to French Agency for Food, Environmental and Occupational Health and Safety (ANSES).

**Table 3.** Dietary sources of omega-3 essential polyunsaturated fatty acids according to ANSES.

	**Content in g/100g**		
	**Alpha-linolenic acid (ALA)**	**Eicosapentaenoic acid (EPA)**	**Docosahexaenoic acid (DHA)**
**Olive oil**	0.65	<0.01	<0.01
**Canola oil**	7.54	0	0
**Soybean oil**	6.89	0	0
**Flaxseed oil**	53.3	0	0
**Fresh walnut**	3.77	<0.01	<0.01
**Sesame seeds**	0.26	0	0
**Flaxseed**	16.7	0	0
**Sardine**	0.47	1.09	1.58
**Tuna**	0.004	0.34	1.08
**Mackerel**	0.19	0.8	1.34

Ciqual French Food Composition Table, 2020.^68^

PUFAs have demonstrated the ability to prevent the development of inflammation by reducing blood levels of inflammatory biomarkers such as interleukin-6 (IL-6), TNF-α, CRP, serum amyloid A, and white blood cell counts.^69^ Several RCTs have explored the efficacy of PUFAs, especially omega-3, in IRDs, and five meta-analyses were found in literature.

The initial meta-analysis assessed the impact of omega-3 (administered at ≥2.7 g/day) for a minimum of 3 months on clinical outcomes in RA patients. This analysis, involving ten RCTs with 183 RA patients and 187 placebo-treated RA controls, demonstrated a significant reduction in non-steroidal anti-inflammatory drug (NSAID) consumption without between-study heterogeneity (I2 = 0%). However, there was no significant improvement in tender joint count and swollen joint count. ^70^

The second meta-analysis, included 20 RCTs examining the effects of orally consumed omega-3 fatty acids over a 3-month period, revealed significant improvements in morning stiffness, tender joint count, ESR and pain scale. Nevertheless, no significant difference was observed in DAS28. ^69^

In more recent meta-analysis, the effects of oral supplementation with both omega-3 and omega-6 PUFAs on all IRDs were evaluated in 30 RCTs. Significant improvements were noted in pain, swollen and tender joint count, DAS28, and HAQ score in IRDs patients receiving PUFA supplementation compared with controls. Additionally, there was a significant decrease in ESR, although not in CRP levels. Subgroup analysis suggested greater improvements with animal-derived PUFAs than vegetable-derived PUFAs, especially with 3- to 6-month supplementation.^71^

Concerning animal-derived PUFAs, a separate meta-analysis focused on the impact of marine oil supplements on pain in patients with arthritis. Results from 22 trials indicated a significant improvement in individuals with RA.^72^

The most recent meta-analysis by Gkiouras K et al., involving twenty-three studies in RA patients, revealed a modest effect in reducing pain, tender and swollen joint count. Sensitivity analyses indicated a small effect in reducing NSAID intake, with CRP reduced by only 0.21 mg/Dl.^73^

In summary, the authors suggest that recommending a diet rich in polyunsaturated fatty acids is advisable, and supplementation could be considered if the patient insists that achieving a balanced diet might be challenging. However, it is important to note that existing studies are of short duration and lack data on the duration of supplementation and sequences. It should be mentioned that excessive supplementation could potentially be toxic.^74,75^ Therefore, the wiser advice would be to prioritise a diet naturally rich in polyunsaturated fatty acids over opting for supplementation.

### Recommendation 6: Probiotics are not currently recommended for managing the disease activity in patients with chronic inflammatory rheumatic diseases (insufficient or contradictory scientific literature data).

In 2002, the World Health Organisation defined probiotics as “living organisms in food and dietary supplements that, upon ingestion, can improve the health of the host beyond their inherent basic nutritional content”.^76^ Patients with IRDs, exhibit altered gut microbiota, known as dysbiosis, leading to increased permeability and enhanced interaction between luminal antigens or bacteria with the host immune system.^77^

In the context of RA, the first meta-analysis, including nine studies, revealed a significant reduction in the pro-inflammatory cytokine IL-6 in the probiotics group compared to the placebo group. However, there was no significant difference in disease activity score between the two groups.^78^

The second meta-analyses, evaluating RA and SpA, included ten RCTs and three meta-analyses. For RA, probiotics showed a significant decrease in CRP concentration. Yet, after excluding trials with a high risk of bias, no significant difference was observed in DAS28.^77^ Furthermore, a more recent meta-analysis, focusing on the safety and efficacy of probiotic supplementation in inflammatory arthritis, showed no statistically significant difference in DAS 28, tender joint count, and swollen joint count between the experimental and control groups.^79^

Among the positive studies for RA, one used strains *L. casei* for 8 weeks,^80^ and the other two used *L. acidophilus*, *L. casei*, and *B. bifidum*, also for 8 weeks, ^81,82^ with a range of 10^8 to 10^9 colony-forming units (CFU) per day.

In SpA, two RCTs assessing probiotics found no significant difference in mean Bath ankylosing spondylitis functional index (BASFI) and Bath Ankylosing Spondylitis Disease Activity Index (BASDAI) after probiotic intervention compared to placebo.^83,84^

In summary, the current data is insufficient to recommend probiotic intake for managing activity in IRDs due to the considerable heterogeneity in studies and the short duration of follow-up.

### Recommendation 7: Spices with anti-inflammatory properties (saffron, cinnamon, garlic, ginger, and curcuma) could have a beneficial effect in patients with rheumatoid arthritis, provided that appropriate doses are used and contraindications are respected, especially for pregnant and breastfeeding women, the elderly, patients with comorbidities, and those on multiple medications. (insufficient data to recommend them for routine practice, and insufficient scientific data for spondyloarthritis and psoriatic arthritis)

Spices hold a long history of medicinal use. In recent years, modern medicine has delved into their potential health benefits, recognising their anti-inflammatory, antioxidant, and analgesic properties, particularly in immune-mediated diseases.^85^

In the context of IRDs, numerous trials, especially in RA, have explored the effects of various spices on RA activity and biological parameters. RCTs investigating cinnamon (four daily capsules, 500 mg)^86^ garlic (1 g powder ≈ 2.5 g fresh garlic/day)^87^**,** and ginger (1.5 g powder/day) demonstrated significant improvements in DAS 28, VAS pain, and tender and swollen joint count.^88^

For saffron, two RCTs were conducted. Hamidi et al. evaluated the effects of 100 mg daily and found significant improvements in DAS 28, pain VAS, tender and swollen joint count.^89^ However, the second RCT evaluated the same saffron dose on newly diagnosed RA patients without previous treatment and found no significant improvement in DAS28 and HAQ scores between the intervention and control groups.^90^

Curcuma emerged as the most studied spice, with doses ranging from 250 to 500 mg per day for 8–12 weeks. A meta-analysis of five RCTs indicated significant improvements in DAS 28, CRP, and ESR. However, no significant decrease was observed in tender and swollen joint count.^91^

It is crucial to note that excessive spice doses can be toxic,^92,93^ and caution should be exercised, especially for pregnant and lactating women, patients with comorbidities, and those on multiple medications. Therefore, the use of spices requires careful consideration, and a benefit-risk evaluation should be undertaken.

In summary, there is a positive signal regarding the efficacy of these spices in RA. However, there is a lack of data comparing the effectiveness of spices when incorporated into food and cooked versus encapsulated spice supplementation. Additionally, the safety of spice supplementation, optimal doses, duration, and treatment sequences need further exploration. Therefore, more RCTs are warranted to thoroughly investigate both the benefits and, most importantly, the safety of spice supplementation.

### Recommendation 8: Scientific data on intermittent fasting or during Ramadan is insufficient or contradictory. However, it is recommended to associate fasting with a healthy and balanced diet.

A number of studies have indicated that intermittent fasting can reduce the levels of various proinflammatory cytokines such as IL-1, TNF, and IL-6.^94^ However, there is limited data regarding the connection between fasting and the activity of autoimmune diseases. In this context, previous research has explored the effects of fasting on RA patients. Two earlier RCTs investigated the impact of fasting (7–10 days), either alone,^95^ or in combination with a gluten-free vegan diet. ^39^.Both studies demonstrated improvements in different RA activity parameters but lacked information on the sustainability of joint effects. It is crucial to note that these studies were conducted in the past, during a period when RA treatment may not have been optimal. In a recent RCT (NutriFast-Study) involving 53 RA patients, the effects of a 7-day fast followed by a plant-based diet were compared to the dietary recommendations of the German Society for Nutrition regarding RA disease activity and cardiovascular risk factors. Results showed improvement in RA disease activity and cardiovascular risk factors in both dietary approaches. However, when compared with a guideline-based anti-inflammatory diet, fasting followed by a plant-based diet showed no benefit in terms of function and disability after 12 weeks.^96^

Concerning Ramadan fasting, a religious practice involving intermittent fasting from sunrise to sunset that aligns with the circadian rhythm, two studies investigated its impact on RA,^97^and PsA.^98^ The first study, a prospective observational study of 240 patients, explored the link between Ramadan fasting and RA activity, revealing a decrease in DAS28 scores for both fasting and non-fasting cohorts. ^97^ For PsA, a multicentre prospective study demonstrated beneficial effects on disease activity scores (BASDAI, enthesitis, and dactylitis) during fasting. Importantly, this benefit was independent of weight loss, which remained stable throughout the study.^98^

In summary, there is currently insufficient data to recommend intermittent fasting or fasting during Ramadan to control inflammatory rheumatic diseases. However, fasting should be accompanied by a balanced diet and adequate hydration to potentially reap benefits. For patients with comorbidities, the decision to fast should be made in consultation between treating physicians and patients.

### Recommendation 9: For patients with chronic inflammatory rheumatic diseases, it is recommended to limit the consumption of sugar-sweetened beverages because of their high sugar content, which can lead to weight gain and increase the risk of cardio-metabolic diseases (insufficient data in the literature to draw conclusions about caffeine and tea. Overall, it is advisable to maintain a good hydration level, giving priority to water).

Previous studies have examined the association between tea and coffee consumption and the risk of RA,^99,100^ but data on the impact of tea and coffee consumption on IRDs activity are quite limited. Concerning tea consumption, to our knowledge, only two studies have been found. The first is a cross-sectional study that explored the potential association of tea consumption with RA activity involving 733 RA patients. The study revealed an inverse association between tea consumption and disease activity in RA patients, and higher tea intake (> 750 mL/day) was associated with lower disease activity in RA.^101^ Similar results were found in a clinical trial, including 120 patients, which revealed that green tea (4–6 cups/day) and exercise provide positive effects on disease activity among RA patients.^102^

Regarding soft sugary drinks, previous studies have also shown their association with the occurrence of overweight and obesity,^103^ metabolic syndrome (MetS), and diabetes.^104,105^ In term of IRDs, a large-scale epidemiological study based on the Nurses’ Health Study initiated in 1976, involving more than 121,000 female registered nurses, it was revealed that consistent intake of sugar-sweetened soda, is linked to an elevated risk of seropositive RA in women, independently of other dietary and lifestyle factors.^106^

In summary, data on tea and coffee consumption are limited, but moderation is advisable. As for sugary soft drinks, given their adverse effects on health, it is recommended to replace them with natural fruit or vegetable juices, which have been shown to be beneficial for health due to their antioxidant and anti-inflammatory properties.^107^

### Recommendation 10: Specific recommendation for Moroccan nutrition. For patients with chronic inflammatory rheumatic diseases, it is advisable to replace white bread with whole grain bread. The consumption of olive oil can be recommended to patients (1–2 tablespoons per day). Red meat consumption should be balanced and adapted according to the patient’s condition and associated comorbidities.

In Morocco, bread holds a central role in daily meals, particularly wheat bread, serving as a primary energy source. Its composition includes a mixture of carbohydrates, proteins, lipids, fibre, minerals, vitamins, and phytochemical compounds.^108^ However, whole grain bread, a component of the MD recommended in both our guidelines and those of the SFR,^8^ has not been exclusively studied in patients with chronic IRDs. A Swedish study evaluating dietary quality in patients with RA found that high dietary quality, characterised by increased intake of fish, shellfish, whole grains, fruits, and vegetables, along with reduced consumption of sausages and sweets, was linked to lower inflammation assessed by CRP and ESR.^109^ Additionally, an anti-inflammatory diet (ITIS diet) designed for RA patients, led by Marta F. Bustamante et al., proposed substituting refined wheat with whole grains like rye, corn, oats, or quinoa.^110^

In terms of comorbidities, a meta-analysis of 64 publications revealed that whole grain intake is associated with a reduced risk of various health diseases, including coronary heart disease, cardiovascular disease, total cancer, all-cause mortality, respiratory diseases, infectious diseases, diabetes, and all non-cardiovascular, non-cancer causes.^111^

The Moroccan population is also known for its consumption of olive oil, especially during breakfast, where many families incorporate it into their cooking. In the MD, olive oil is favoured as a source of vegetable lipids, recommended at a daily intake. Research on cooking with olive oil under various conditions suggests that it generally shows good thermal resistance compared to other vegetable oils. However, to preserve all of its bio-active components, it is advisable to minimise heating time as much as possible. Therefore, using olive oil as a final seasoning in fresh salads, soups, or more elaborate dishes is recommended.^112^

In terms of olive oil consumption, a trial exploring supplementary formulations of olive oil, figs, and olives showed improvements in disease activity, although not statistically significant.^113^ Another cross-sectional study focusing on two food groups, olive oil and nuts, revealed a favourable effect on disease activity, significant only for olive oil.^114^ Therefore, the authors suggest recommending whole grain bread, such as wheat bread, over white bread, and increasing the consumption of olive oil as a source of vegetable lipids in patients with IRDs.

There is a clear shift in the Moroccan population from a MD towards a more Westernized diet, characterised by an increasing consumption of red and processed meat during the last decades.^115^ However, in the MD, reducing red meat consumption is advised, especially concerning comorbidities. A large-scale study with 4,778 participants demonstrated that meat consumption is associated with several metabolic markers, providing insight into the harmful effects of red meat consumption on cardiovascular disease. ^116^ The ITIS model also recommends decreasing red meat consumption due to its high levels of choline, a precursor to the inflammatory metabolite TMAO, and saturated fatty acids.^110^

### Recommendation 11: Specific recommendation for Moroccan nutrition: Scientific data on flaxseed and nigella, widely used in traditional Moroccan medicine, is insufficient or contradictory to recommend them to patients with chronic inflammatory rheumatic diseases.

Flaxseed (linseed), an edible oil seed/grain, stands out as the richest plant source of ALA, constituting 50%– 62% of flaxseed oil or 22% of whole flaxseed. ALA is an omega-3 (n-3) PUFA.^117^ In the recommendation N°5, the benefits of omega-3 in the activity of IRDs, especially RA, have been emphasised. However, studies exclusively focusing on flaxseed are limited. Two RCTs were identified. The first RCT demonstrated no improvement in various parameters of RA activity.^118^ However, the second trial, involving 120 RA patients, revealed that Flaxseed (30g/day) decreased DAS28, pain severity, and morning stiffness.^119^ Two meta-analyses assessed the effectiveness of flaxseed on CRP and inflammatory cytokines in adults, the first found no significant decrease in CRP levels,^117^ while the second revealed a significant reduction in concentrations of CRP and IL-6.^120^ Black cumin (Nigella sativa) holds significant value in traditional medicine. It exhibits anti-inflammatory, antiallergic, antitumor, hypoglycaemic, antioxidant, hypotensive, hypolipidemic, immunomodulatory, nephroprotective, diuretic, anti-ulcer, and hepatoprotective effects.^121^ Despite these beneficial effects, there are limited clinical studies on black cumin and IRDs, with three RCTs conducted in RA. Two of these evaluated black cumin oil capsules (1 g/day) for 8 weeks, demonstrating a significant decrease in DAS28 in both studies.^122,123^ The third RCT explored a low-calorie diet combined with 3 g/day of Nigella sativa oil, which lowered levels of TNF and CRP compared to the placebo group.^124^

In conclusion, although flaxseed and Nigella sativa could have beneficial effects, the available data are insufficient to warrant their recommendation for patients with IRDs. Patients who choose to use these supplements should exercise caution, strictly adhere to recommended doses, and consider avoiding them during pregnancy, in the elderly, in patients with comorbidities, and in those taking multiple medications.

## DISCUSSION

Five general principles and eleven recommendations were established, leading to a broad consensus within the group. This consensus is expected to enhance the feasibility and practicability of the discussed principles and recommendations.

In the context of chronic IRDs, non-pharmacological treatment is a fundamental aspect of management, incorporating dietary interventions. However, it should not replace pharmacological treatment but rather support it to achieve optimal effectiveness, always adhering to the “treat-to-target” approach. Nutrition, therefore, becomes an integral part of overall patient care and should be accompanied by adapted physical activity. These recommendations encourage patients to integrate nutrition into their daily lives and prompt treating physicians to discuss it with their patients, utilising a valid tool based on robust literature data (second general principle).

Eleven recommendations were formulated, including two specific recommendations to Moroccan nutrition. Positive recommendations involved weight loss, a Mediterranean-type diet, and a diet rich in omega-3 or supplementation of essential PUFAs. For Moroccan nutrition, it is advised to replace white bread with whole grain bread and recommend increasing the consumption of olive oil, preferably within a MD. This aligns with the dietary recommendations of the SFR, which proposed weight loss in obese or overweight patients to control IRDs activity, the MD and supplementation of PUFAs. In our recommendations, we lean towards a diet rich in omega-3 rather than supplementation due to insufficient information on duration, sequencing, and the difficulty of monitoring to avoid toxicity. The recommendation specifies that supplementation could be considered for patients interested in additional supplementation alongside pharmacological treatment. Additionally, it is recommended to limit the consumption of sugar-sweetened beverages to prevent weight gain and to reduce the risk of cardio-metabolic diseases.

Regarding negative recommendations, exclusion diets such as gluten-free (except in cases of associated celiac disease), vegetarian, and dairy-free diets are not recommended due to insufficient data for symptomatic relief. However, if a patient is on an exclusion diet, support and encouragement are necessary, especially if positive results are observed. Additionally, in the SFR recommendations, a gluten-free diet was not recommended to manage IRDs activity. A recommendation was designed for the gluten-free diet due to its popularity in France, unlike in Morocco where it is less common. Vitamin or trace element supplementation is not recommended systematically, but supplementation is essential in the case of deficiency. However, more RCTs are necessary to evaluate certain supplements, notably coenzyme Q10, which could have a beneficial effect in RA patients. Probiotics are not currently recommended due to contradictory and insufficient data. Some recommendations are considered ‘borderline,’ such as the beneficial effect of spices with anti-inflammatory properties. However, there is insufficient data to recommend them for routine practice.

In the Moroccan context, data are insufficient to recommend Ramadan fasting, but the practice of fasting should be associated with a balanced diet. It is also advisable to encourage the limitation of red meat consumption in daily life or during specific events like ‘Aid el Adha,’ with reasonable intake, especially for older individuals with cardio-metabolic comorbidities.

Certain traditionally used grains, like flaxseed and black cumin, could have beneficial effects, but there is insufficient solid data to recommend them for routine use. If patients wish to take them, guidance on recommended doses is essential.

Our recommendations are subject to several limitations. First, a considerable number of studies have not adhered to robust methodologies, which limits the strength of the conclusions. Second, there are substantial inter-individual differences in patients’ sensitivity to dietary factors, particularly given the significant variability in gut microbiota composition. Third, circa-dian rhythms have profound effects on biological processes, including the cell cycle and gene expression, and play a critical role in inflammatory and immune responses. Recently, increasing attention has been directed towards the relationship between diet—both its quality and timing—and circadian rhythms.^125,126^ This important aspect warrants consideration and integration into future dietary recommendations.

In summary, our recommendations align with the initial international nutrition recommendations established by the French Society for Rheumatology, endorsing weight loss, a Mediterranean-type diet, and a diet rich in omega-3 or supplementation of essential polyunsaturated fatty acids in practice.

## CONCLUSION

These recommendations represent the first national and the second international nutrition recommendations for patients with IRDs. The ultimate objective of this work was to establish a validated and easy-to-use tool that enhances the overall care of patients affected by IRDs. Subsequent updates will be contemplated, acknowledging the dynamic nature of the literature in this field.

## CONFLICT OF INTEREST

The authors declare no conflict of interest.

## References

[1] World Health Organization. WHO technical report series: Rheumatic diseases. Geneva: World Health Organization; 1992. Report No.: 816. 1561791

[2] Ajeganova S Huizinga T. Sustained remission in rheumatoid arthritis: Latest evidence and clinical considerations. Adv Musculoskelet Dis 2017;9:249–62. 10.1177/1759720X17720366 PMC5613855 28974987

[3] Daien C Hua C Gaujoux-Viala C Cantagrel A Dubremetz M Dougados M Update of French society for rheumatology recommendations for managing rheumatoid arthritis. Joint Bone Spine 2019;86(2):135–50. 30315988 10.1016/j.jbspin.2018.10.002

[4] Wendling D Lukas C Prati C Claudepierre P Gossec L Goupille P 2018 update of French Society for Rheumatology (SFR) recommendations about the everyday management of patients with spondyloarthritis. Joint Bone Spine 2018;85(3):275–84. 29407043 10.1016/j.jbspin.2018.01.006

[5] Bentaleb I Oulkadi L Jaouad N Maghraoui AE Niamane R Bouchti IE Recommendations of the Moroccan Society of Rheumatology (SMR) for Diagnostic Management of Spondyloarthritis (SpA) and Psoriatic Arthritis (PsA). Mediterr J Rheumatol 2023;34(3):302–14. 37941856 10.31138/mjr.230929rtm PMC10628882

[6] Wang Y Wei J Zhang W Doherty M Zhang Y Xie H Gut dysbiosis in rheumatic diseases: A systematic review and meta-analysis of 92 observational studies. EBioMedicine 2022; 80:104055. 35594658 10.1016/j.ebiom.2022.104055 PMC9120231

[7] Semerano L Julia C Aitisha O Boissier MC. Nutrition and chronic inflammatory rheumatic disease. Joint Bone Spine 2017;84(5):547–52. 27914804 10.1016/j.jbspin.2016.10.003

[8] Daien C Czernichow S Letarouilly JG Nguyen Y Sanchez P Sigaux J Dietary recommendations of the French Society for Rheumatology for patients with chronic inflammatory rheumatic diseases. Joint Bone Spine 2022;89(2):105319. 34902577 10.1016/j.jbspin.2021.105319

[9] Berrichi I Taik FZ Takhrifa N Haddani F Berrada K Abourazzak FZ. Les patients marocains discutentils avec leurs rhumatologues à propos du régime alimentaire comme partie de la prise en charge de leur RIC? [Abstract] Rev Rhum 2022;89(Suppl 1).

[10] van der Heijde D Aletaha D Carmona L Edwards CJ Kvien TK Kouloumas M 2014 Update of the EULAR standardised operating procedures for EULAR-endorsed recommendations. Ann Rheum Dis 2015;74(1):8–13. 25261577 10.1136/annrheumdis-2014-206350 PMC4283681

[11] Liberati A Altman DG Tetzlaff J Mulrow C Gøtzsche PC Ioannidis JP The PRISMA statement for reporting systematic reviews and meta-analyses of studies that evaluate health care interventions: explanation and elaboration. J Clin Epidemiol 2009;62(10): e1–34. 19631507 10.1016/j.jclinepi.2009.06.006

[12] Oxford Centre for Evidence-based Medicine Levels of Evidence. March 2009. Available from: https://www.cebm.ox.ac.uk/resources/levels-of-evidence/oxford-centre-for-evidence-based-medicine-levels-of-evidence-march-2009

[13] Available from: https://globalnutritionreport.org/resources/nutrition-profiles/africa/northern-africa/morocco/

[14] Ranganath VK La Cava A Vangala S Brook J Kermani TA Furst DE Improved outcomes in rheumatoid arthritis with obesity after a weight loss intervention: randomized trial. Rheumatology (Oxford) 2023;62(2):565–74. 35640116 10.1093/rheumatology/keac307

[15] di Minno MN Peluso R Iervolino S Lupoli R Russolillo A Scarpa R Obesity and the prediction of minimal disease activity: a prospective study in psoriatic arthritis. Arthritis Care Res (Hoboken) 2013;65(1):141–7. 22514189 10.1002/acr.21711

[16] Kreps DJ Halperin F Desai SP Zhang ZZ Losina E Olson AT Association of weight loss with improved disease activity in patients with rheumatoid arthritis: A retrospective analysis using electronic medical record data. Int J Clin Rheumatol 2018;13(1):1–10. 10.4172/1758-4272.1000154 PMC5875117 29606976

[17] Weijers JM Müskens WD van Riel PLCM. Effect of significant weight loss on disease activity: reason to implement this nonpharmaceutical intervention in daily clinical practice. RMD Open 2021;7(1):e001498. 33504577 10.1136/rmdopen-2020-001498 PMC7843325

[18] Egeberg A Sørensen JA Gislason GH Knop FK Skov L. Incidence and Prognosis of Psoriasis and Psoriatic Arthritis in Patients Undergoing Bariatric Surgery. JAMA Surg 2017;152(4):344–9. 28002543 10.1001/jamasurg.2016.4610

[19] Ortolan A Felicetti M Lorenzin M Cozzi G Ometto F Striani G The impact of diet on disease activity in spondyloarthritis: A systematic literature review. Joint Bone Spine 2023;90(2):105476. 36404571 10.1016/j.jbspin.2022.105476

[20] Bach-Faig A Berry EM Lairon D Reguant J Trichopoulou A Dernini S Mediterranean diet pyramid today. Science and cultural updates. Public Health Nutr 2011;14(12A):2274–84. 22166184 10.1017/S1368980011002515

[21] Davis C Bryan J Hodgson J Murphy K. Definition of the Mediterranean Diet; a Literature Review. Nutrients 2015;7(11):9139–53. 26556369 10.3390/nu7115459 PMC4663587

[22] Widmer RJ Flammer AJ Lerman LO Lerman A. The Mediterranean diet, its components, and cardiovascular disease. Am J Med 2015;128:229–38. 25447615 10.1016/j.amjmed.2014.10.014 PMC4339461

[23] Sköldstam L Hagfors L Johansson G. An experimental study of a Mediterranean diet intervention for patients with rheumatoid arthritis. Ann Rheum Dis 2003;62(3):208–14. 12594104 10.1136/ard.62.3.208 PMC1754463

[24] Hansen GV Nielsen L Kluger E Thysen M Emmertsen H Stengaard-Pedersen K Nutritional status of Danish rheumatoid arthritis patients and effects of a diet adjusted in energy intake, fish-meal, and antioxidants. Scand J Rheumatol 1996;25(5):325–30. 8921927 10.3109/03009749609104066

[25] McKellar G Morrison E McEntegart A Hampson R Tierney A Mackle G A pilot study of a Mediterranean-type diet intervention in female patients with rheumatoid arthritis living in areas of social deprivation in Glasgow. Ann Rheum Dis 2007. 10.1136/ard.2006.065151 PMC1955146 17613557

[26] Vadell AKE Bärebring L Hulander E. Anti-inflammatory Diet In Rheumatoid Arthritis (ADIRA) – a randomized, controlled crossover trial indicating effects on disease activity. Am J Clin Nutr 2020. 10.1093/ajcn/nqaa019 PMC7266686 32055820

[27] Sadeghi A Tabatabaiee M Mousavi M Abdollahi S Jalili N. Dietary Pattern or Weight Loss: Which One Is More Important to Reduce Disease Activity Score in Patients with Rheumatoid Arthritis? A Randomized Feeding Trial. Int J Clin Pract 2022;6004916. 35685522 10.1155/2022/6004916 PMC9159180

[28] García-Morales JM Lozada-Mellado M Hinojosa-Azaola A Llorente L Ogata-Medel M Pineda-Juárez JA Effect of a Dynamic Exercise Program in Combination With Mediterranean Diet on Quality of Life in Women With Rheumatoid Arthritis. J Clin Rheumatol 2020;26(7S):S116–S122. 31145222 10.1097/RHU.0000000000001064

[29] Papandreou P Gioxari A Daskalou E Grammatikopoulou MG Skouroliakou M Bogdanos DP. Mediterranean Diet and Physical Activity Nudges versus Usual Care in Women with Rheumatoid Arthritis: Results from the MADEIRA Randomized Controlled Trial. Nutrients 2023;15(3):676. 36771382 10.3390/nu15030676 PMC9919932

[30] Ometto F Ortolan A Farber D Lorenzin M Dellamaria G Cozzi G Mediterranean diet in axial spondyloarthritis: an observational study in an Italian monocentric cohort. Arthritis Res Ther 2021;23(1):219. 34416917 10.1186/s13075-021-02600-0 PMC8377333

[31] Alawadhi B Alsaber A Shatawan I Al-Herz A Setiya P Saleh K Adherence to the Mediterranean diet is associated with a reduced DAS28 index among patients with rheumatoid arthritis: Case study from KRRD. Int J Rheum Dis 2023; Oct 2. 10.1111/1756-185X.14928 37784239

[32] Charneca S Ferro M Vasques J Carolino E Martins-Martinho J Duarte-Monteiro AM The Mediterranean diet, and not dietary inflammatory index, is associated with rheumatoid arthritis disease activity, the impact of disease and functional disability. Eur J Nutr 2023;62(7):2827–2839. 37355497 10.1007/s00394-023-03196-8

[33] Zhan Y Yang Z Liu Y Zhan F Lin S. Interaction between rheumatoid arthritis and Mediterranean diet on the risk of cardiovascular disease for the middle-aged and elderly from National Health and Nutrition Examination Survey (NHANES). BMC Public Health 2023;23(1):620. 37003993 10.1186/s12889-023-15478-1 PMC10067192

[34] Delgado-Lista J Alcala-Diaz JF Torres-Peña JD Quintana-Navarro GM Fuentes F Garcia-Rios A Long-term secondary prevention of cardiovascular disease with a Mediterranean diet and a low-fat diet (CORDIOPREV): a randomised controlled trial. Lancet 2022;399(10338):1876–85. 35525255 10.1016/S0140-6736(22)00122-2

[35] Hafström I Ringertz B Spångberg A von Zweigbergk L Brannemark S Nylander I A vegan diet free of gluten improves the signs and symptoms of rheumatoid arthritis: the effects on arthritis correlate with a reduction in antibodies to food antigens. Rheumatology 2001;40:1175–9. 11600749 10.1093/rheumatology/40.10.1175

[36] Elkan AC Sjöberg B Kolsrud B Ringertz B Hafström I Frostegård J. Gluten-free vegan diet induces decreased LDL and oxidized LDL levels and raised atheroprotective natural antibodies against phosphorylcholine in patients with rheumatoid arthritis: a randomized study. Arthritis Res Ther 2008;10(2):R34. 18348715 10.1186/ar2388 PMC2453753

[37] Lidón AC Patricia ML Vinesh D Marta MS. Evaluation of Gluten Exclusion for the Improvement of Rheumatoid Arthritis in Adults. Nutrients 2022;14(5396). 10.3390/nu14245396 PMC9783934 36558555

[38] Dürholz K Hofmann J Iljazovic A Häger J Lucas S Sarter K Dietary Short-Term Fiber Interventions in Arthritis Patients Increase Systemic SCFA Levels and Regulate Inflammation. Nutrients 2020;12:3207. 33092271 10.3390/nu12103207 PMC7589100

[39] Kjeldsen-Kragh J Haugen M Borchgrevink CF Laerum E Eek M Mowinkel P Controlled trial of fasting and one-year vegetarian diet in rheumatoid arthritis. Lancet 1991;338:899–902. 1681264 10.1016/0140-6736(91)91770-u

[40] Nenonen MT Helve TA Rauma AL Hänninen OO. Uncooked, lactobacilli-rich, vegan food and rheumatoid arthritis. Br J Rheumatol 1998;37(3):274–81. 9566667 10.1093/rheumatology/37.3.274

[41] Adam O Beringer C Kless T Lemmen C Adam A Wiseman M Anti-inflammatory effects of a low arachidonic acid diet and fish oil in patients with rheumatoid arthritis. Rheumatol Int 2003;23(1):27–36. 12548439 10.1007/s00296-002-0234-7

[42] McDougall J Bruce B Spiller G Westerdahl J McDougall M. Effects of a very low-fat, vegan diet in subjects with rheumatoid arthritis. J Altern Complement Med 2002;8(1):71–75. 11890437 10.1089/107555302753507195

[43] Labonte ME Couture P Richard C Desroches S Lamarche B. Impact of dairy products on biomarkers of inflammation: a systematic review of randomized controlled nutritional intervention studies in overweight and obese adults. Am J Clin Nutr 2013;97:706–717. 23446894 10.3945/ajcn.112.052217

[44] Gioia C Lucchino B Tarsitano MG Iannuccelli C Di Franco M. Dietary Habits and Nutrition in Rheumatoid Arthritis: Can Diet Influence Disease Development and Clinical Manifestations? Nutrients 2020;12(5):1456. 32443535 10.3390/nu12051456 PMC7284442

[45] Guagnano MT D’Angelo C Caniglia D Di Giovanni P Celletti E Sabatini E Improvement of Inflammation and Pain after Three Months’ Exclusion Diet in Rheumatoid Arthritis Patients. Nutrients 2021;13(10):3535. 34684536 10.3390/nu13103535 PMC8539601

[46] Companys J Pla-Pagà L Calderón-Pérez L Llauradó E Solà R Pedret A Fermented Dairy Products, Probiotic Supplementation, and Cardiometabolic Diseases: A Systematic Review and Meta-analysis. Adv Nutr 2020;11(4):834–63. 32277831 10.1093/advances/nmaa030 PMC7360468

[47] Franco AS Freitas TQ Bernardo WM Pereira RMR. Vitamin D supplementation and disease activity in patients with immune-mediated rheumatic diseases: A systematic review and meta-analysis. Medicine (Baltimore) 2017;96(23):e7024. 28591033 10.1097/MD.0000000000007024 PMC5466211

[48] Nguyen Y Sigaux J Letarouilly JG Sanchez P Czernichow S Flipo RM Efficacy of Oral Vitamin Supplementation in Inflammatory Rheumatic Disorders: A Systematic Review and Meta-Analysis of Randomized Controlled Trials. Nutrients 2020;13(1):107. 33396851 10.3390/nu13010107 PMC7824121

[49] Guan Y Hao Y Guan Y Bu H Wang H. The Effect of Vitamin D Supplementation on Rheumatoid Arthritis Patients: A Systematic Review and Meta-Analysis. Front Med 2020;7:596007. 10.3389/fmed.2020.596007 PMC7661491 33195358

[50] Kou H Qing Z Guo H Zhang R Ma J. Effect of vitamin E supplementation in rheumatoid arthritis: a systematic review and meta-analysis. Eur J Clin Nutr 2023;77(2):166–72. 35468933 10.1038/s41430-022-01148-9

[51] Kolahi S Pourghassem Gargari B Mesgari Abbasi M Asghari Jafarabadi M Ghamarzad Shishavan N. Effects of phylloquinone supplementation on lipid profile in women with rheumatoid arthritis: a double-blind placebo-controlled study. Nutr Res Pract 2015;9(2):186–91. 25861426 10.4162/nrp.2015.9.2.186 PMC4388951

[52] Shishavan NG Gargari BP Kolahi S Hajialilo M Jafarabadi MA Javadzadeh Y. Effects of Vitamin K on Matrix Metalloproteinase-3 and Rheumatoid Factor in Women with Rheumatoid Arthritis: A Randomized, Double-Blind, Placebo-Controlled Trial. J Am Coll Nutr 2016;35(5):392–8. 26156560 10.1080/07315724.2015.1026004

[53] Morgan SL Baggott JE Vaughn WH Austin JS Veitch TA Lee JY Supplementation with folic acid during methotrexate therapy for rheumatoid arthritis. A double-blind, placebo-controlled trial. Ann Intern Med 1994;121(11):833–41. 7978695 10.7326/0003-4819-121-11-199412010-00002

[54] Stamp LK O’Donnell JL Frampton C Drake J Zhang M Barclay M A Pilot Randomized Controlled Double-Blind Trial of High- Versus Low-Dose Weekly Folic Acid in People With Rheumatoid Arthritis Receiving Methotrexate. J Clin Rheumatol 2019;25(7):284–7. 30001258 10.1097/RHU.0000000000000848

[55] Fairweather-Tait SJ Bao Y Broadley MR Collings R Ford D Hesketh JE Selenium in human health and disease. Antioxid Redox Signal 2011;14(7):1337–83. 20812787 10.1089/ars.2010.3275

[56] Turrubiates-Hernández FJ Márquez-Sandoval YF González-Estevez G Reyes-Castillo Z Muñoz-Valle JF. The Relevance of Selenium Status in Rheumatoid Arthritis. Nutrients 2020;12(10):3007. 33007934 10.3390/nu12103007 PMC7601319

[57] Tarp U Overvad K Thorling EB Graudal H Hansen JC. Selenium treatment in rheumatoid arthritis. Scand J Rheumatol 1985;14(4):364–8. 3909379 10.3109/03009748509102039

[58] Peretz A Neve J Duchateau J Famaey JP. Adjuvant treatment of recent onset rheumatoid arthritis by selenium supplementation: preliminary observations. Br J Rheumatol 1992;31(4):281–2. 1555045 10.1093/rheumatology/31.4.281

[59] Heinle K Adam A Gradl M Wiseman M Adam O. Selenkonzentration in den Erythrozyten bei Patienten mit rheumatoider Arthritis. Med Klin 1997;92:29–31. 10.1007/BF03041958 9417493

[60] Peretz A Siderova V Nève J. Selenium supplementation in rheumatoid arthritis investigated in a double-blind, placebo-controlled trial. Scand J Rheumatol 2001;30(4):208–12. 11578015 10.1080/030097401316909549

[61] Simkin PA. Oral zinc sulphate in rheumatoid arthritis. Lancet 1976;2:539–42. 60622 10.1016/s0140-6736(76)91793-1

[62] Mattingly PC Mowat AG. Zinc sulphate in rheumatoid arthritis. Ann Rheum Dis 1982;41:456–7. 6751243 10.1136/ard.41.5.456 PMC1001021

[63] Freire de Carvalho J Skare T. Coenzyme Q10 supplementation in rheumatic diseases: A systematic review. Clin Nutr ESPEN 2024; 59:63–9. 38220408 10.1016/j.clnesp.2023.11.016

[64] Nachvak SM Alipour B Mahdavi AM Aghdashi MA Abdollahzad H Pasdar Y Effects of coenzyme Q10 supplementation on matrix metalloproteinases and DAS-28 in patients with rheumatoid arthritis: a randomized, double-blind, placebo-controlled clinical trial. Clin Rheumatol 2019;38(12):3367–74. 31392559 10.1007/s10067-019-04723-x

[65] Abdollahzad H Aghdashi MA Asghari Jafarabadi M Alipour B. Effects of Coenzyme Q10 Supplementation on Inflammatory Cytokines (TNF-α, IL-6) and Oxidative Stress in Rheumatoid Arthritis Patients: A Randomized Controlled Trial. Arch Med Res 2015;46(7):527–33. 26342738 10.1016/j.arcmed.2015.08.006

[66] Rastmanesh R Abargouei AS Shadman Z Ebrahimi AA Weber CE. A pilot study of potassium supplementation in the treatment of hypokalemic patients with rheumatoid arthritis: a randomized, double-blinded, placebo-controlled trial. J Pain 2008;9(8):722–31. 18468955 10.1016/j.jpain.2008.03.006

[67] Kharaeva Z Gostova E De Luca C Raskovic D Korkina L. Clinical and biochemical effects of coenzyme Q10, vitamin E, and selenium supplementation to psoriasis patients. Nutrition 2009;25(3):295–302. 19041224 10.1016/j.nut.2008.08.015

[68] ANSES. Ciqual French Food Composition Table. 2020. Available online at: https://ciqual.anses.fr/.

[69] Gioxari A Kaliora AC Marantidou F Panagiotakos DP. Intake of ω-3 polyunsaturated fatty acids in patients with rheumatoid arthritis: A systematic review and meta-analysis. Nutrition 2018;45:114–24.e4. 28965775 10.1016/j.nut.2017.06.023

[70] Lee YH Bae SC Song GG. Omega-3 polyunsaturated fatty acids and the treatment of rheumatoid arthritis: a meta-analysis. Arch Med Res 2012;43(5):356–62. 22835600 10.1016/j.arcmed.2012.06.011

[71] Sigaux J Mathieu S Nguyen Y Sanchez P Letarouilly JG Soubrier M Impact of type and dose of oral polyunsaturated fatty acid supplementation on disease activity in inflammatory rheumatic diseases: a systematic literature review and meta-analysis. Arthritis Res Ther 2022;24(1):100. 35526074 10.1186/s13075-022-02781-2 PMC9077862

[72] Senftleber NK Nielsen SM Andersen JR Bliddal H Tarp S Lauritzen L Marine oil supplements for arthritis pain: a systematic review and meta-analysis of randomized trials. Nutrients 2017;9(1):42. 28067815 10.3390/nu9010042 PMC5295086

[73] Gkiouras K Grammatikopoulou MG Myrogiannis I Papamitsou T Rigopoulou EI Sakkas LI Efficacy of n-3 fatty acid supplementation on rheumatoid arthritis’ disease activity indicators: a systematic review and meta-analysis of randomized placebo-controlled trials. Crit Rev Food Sci Nutr 2024;64(1):16–30. 35900212 10.1080/10408398.2022.2104210

[74] Clarke J Herzberg G Peeling J Buist R Corbett D. Dietary supplementation of omega-3 polyunsaturated fatty acids worsens forelimb motor function after intracerebral hemorrhage in rats. Exp Neurol 2005;191(1):119–27. 15589518 10.1016/j.expneurol.2004.09.003

[75] Carbone A Psaltis PJ Nelson AJ Metcalf R Richardson JD Weightman M Dietary omega-3 supplementation exacerbates left ventricular dysfunction in an ovine model of anthracycline-induced cardiotoxicity. J Card Fail 2012;18(6):502–11. 22633309 10.1016/j.cardfail.2012.03.005

[76] Food and Agriculture Organization; World Health Organization. Evaluation of health and nutritional properties of probiotics in food including powder milk with live acid bacteria. Report of a joint FAO/WHO Expert Consultation; FAO Food and Nutrition Paper; World Health Organization: Cordoba, Argentina; 2001;85:5–35.

[77] Sanchez P Letarouilly JG Nguyen Y Sigaux J Barnetche T Czernichow S Efficacy of probiotics in rheumatoid arthritis and spondyloarthritis: a systematic review and meta-analysis of randomized controlled trials. Nutrients 2022;14(2):354. 35057535 10.3390/nu14020354 PMC8779560

[78] Mohammed AT Khattab M Ahmed AM Turk T Sakr N Khalil AM The therapeutic effect of probiotics on rheumatoid arthritis: a systematic review and meta-analysis of randomized control trials. Clin Rheumatol 2017;36(12):2697–707. 28914373 10.1007/s10067-017-3814-3

[79] Zeng L Deng Y He Q Yang K Li J Xiang W Safety and efficacy of probiotic supplementation in 8 types of inflammatory arthritis: a systematic review and meta-analysis of 34 randomized controlled trials. Front Immunol 2022;13:961325. 36217542 10.3389/fimmu.2022.961325 PMC9547048

[80] Alipour B Homayouni-Rad A Vaghef-Mehrabany E Sharif SK Vaghef-Mehrabany L Asghari-Jafarabadi M Effects of Lactobacillus casei supplementation on disease activity and inflammatory cytokines in rheumatoid arthritis patients: a randomized double-blind clinical trial. Int J Rheum Dis 2014;17(5):519–27. 24673738 10.1111/1756-185X.12333

[81] Zamani B Golkar HR Farshbaf S Emadi-Baygi M Tajabadi-Ebrahimi M Jafari P Clinical and metabolic response to probiotic supplementation in patients with rheumatoid arthritis: a randomized, double-blind, placebo-controlled trial. Int J Rheum Dis 2016;19(9):869–79. 27135916 10.1111/1756-185X.12888

[82] Zamani B Farshbaf S Golkar HR Bahmani F Asemi Z. Synbiotic supplementation and the effects on clinical and metabolic responses in patients with rheumatoid arthritis: a randomised, double-blind, placebo-controlled trial. Br J Nutr 2017;117(8):1095–102. 28490394 10.1017/S000711451700085X

[83] Brophy S Burrows CL Brooks C Gravenor MB Siebert S Allen SJ. Internet-based randomised controlled trials for the evaluation of complementary and alternative medicines: probiotics in spondyloarthropathy. BMC Musculoskelet Disord 2008;9:4. 18190710 10.1186/1471-2474-9-4 PMC2241591

[84] Jenks K Stebbings S Burton J Schultz M Herbison P Highton J. Probiotic therapy for the treatment of spondyloarthritis: a randomized controlled trial. J Rheumatol 2010;37(10):2118–25. 20716665 10.3899/jrheum.100193

[85] Charneca S Hernando A Costa-Reis P Guerreiro CS. Beyond seasoning—The role of herbs and spices in rheumatic diseases. Nutrients 2023;15:2812. 37375716 10.3390/nu15122812 PMC10300823

[86] Shishehbor F Rezaeyan Safar M Rajaei E Haghighizadeh MH. Cinnamon consumption improves clinical symptoms and inflammatory markers in women with rheumatoid arthritis. J Am Coll Nutr 2018;37:685–90. 10.1080/07315724.2018.1460733 29722610

[87] Moosavian SP Paknahad Z Habibagahi Z Maracy M. The effects of garlic (Allium sativum) supplementation on inflammatory biomarkers, fatigue, and clinical symptoms in patients with active rheumatoid arthritis: a randomized, double-blind, placebo-controlled trial. Phytother Res 2020;34:2953–62. 32478922 10.1002/ptr.6723

[88] Aryaeian N Shahram F Mahmoudi M Tavakoli H Yousefi B Arablou T. The effect of ginger supplementation on some immunity and inflammation intermediate genes expression in patients with active rheumatoid arthritis. Gene 2019;698:179–85. 30844477 10.1016/j.gene.2019.01.048

[89] Hamidi Z Aryaeian N Abolghasemi J Shirani F Hadidi M Fallah S The effect of saffron supplement on clinical outcomes and metabolic profiles in patients with active rheumatoid arthritis: A randomized, double-blind, placebo-controlled clinical trial. Phytother Res 2020;34:1650–58. 32048365 10.1002/ptr.6633

[90] Sahebari M Heidari H Nabavi S Khodashahi M Rezaieyazdi Z Dadgar Moghaddam M A double-blind placebo-controlled randomized trial of oral saffron in the treatment of rheumatoid arthritis. Avicenna J Phytomed 2021;11:332–42. 34290965 10.22038/AJP.2020.17280 PMC8264227

[91] Zeng L Yang T Yang K Yu G Li J Xiang W Efficacy and safety of curcumin and Curcuma longa extract in the treatment of arthritis: A systematic review and meta-analysis of randomized controlled trials. Front Immunol 2022;13:891822. 35935936 10.3389/fimmu.2022.891822 PMC9353077

[92] Gonçalves L Fernandes T Bernardo M Assessment of human health risk of toxic elements due to cinnamon ingestion in the diet. Biol Trace Elem Res 2019;189:313–24. 30191399 10.1007/s12011-018-1473-0

[93] Balaji S Chempakam B. Toxicity prediction of compounds from turmeric (Curcuma longa L). Food Chem Toxicol 2010;48(10):2951–9. 20667459 10.1016/j.fct.2010.07.032

[94] Adawi M Watad A Brown S Aazza K Aazza H Zouhir M Ramadan fasting exerts immunomodulatory effects: Insights from a systematic review. Front Immunol 2017;8:1144. 29230208 10.3389/fimmu.2017.01144 PMC5712070

[95] Udén AM Trang L Venizelos N Palmblad J. Neutrophil functions and clinical performance after total fasting in patients with rheumatoid arthritis. Ann Rheum Dis 1983;42(1):45–51. 10.1136/ard.42.1.45 PMC1001058 6338840

[96] Hartmann AM Dell’Oro M Spoo M Fischer JM Steckhan N Jeitler M To eat or not to eat—an exploratory randomized controlled trial on fasting and plant-based diet in rheumatoid arthritis (NutriFast-Study). Front Nutr 2022;9:1030380. 36407522 10.3389/fnut.2022.1030380 PMC9667053

[97] Siddique S Imran Y Afzal MN Malik U. Effect of Ramadan fasting on disease activity in patients with rheumatoid arthritis presenting in tertiary care hospital. Pak J Med Sci 2020;36(5):1032–5. 32704284 10.12669/pjms.36.5.2099 PMC7372658

[98] Adawi M Damiani G Bragazzi NL Bridgewood C Pacifico A Conic RRZ The impact of intermittent fasting (Ramadan fasting) on psoriatic arthritis disease activity, enthesitis, and dactylitis: A multicentre study. Nutrients 2019;11(3):601. 30871045 10.3390/nu11030601 PMC6471071

[99] Asoudeh F Dashti F Jayedi A Hemmati A Fadel A Mohammadi H. Caffeine, coffee, tea and risk of rheumatoid arthritis: Systematic review and dose-response meta-analysis of prospective cohort studies. Front Nutr 2022;9:822557. 35223954 10.3389/fnut.2022.822557 PMC8866764

[100] Lee YH Bae SC Song GG. Coffee or tea consumption and the risk of rheumatoid arthritis: A meta-analysis. Clin Rheumatol 2014;33(11):1575–83. 24763752 10.1007/s10067-014-2631-1

[101] Jin J Li J Gan Y Liu J Zhao X Chen J Tea consumption is associated with decreased disease activity of rheumatoid arthritis in a real-world, large-scale study. Ann Nutr Metab 2020;76(1):54–61. 32182619 10.1159/000505952

[102] Alghadir AH Gabr SA Al-Eisa ES. Green tea and exercise interventions as nondrug remedies in geriatric patients with rheumatoid arthritis. J Phys Ther Sci 2016;28(10):2820–9. 27821943 10.1589/jpts.28.2820 PMC5088134

[103] Hu H Song J MacGregor GA He FJ. Consumption of soft drinks and overweight and obesity among adolescents in 107 countries and regions. JAMA Netw Open 2023;6(7): e2325158. 37486630 10.1001/jamanetworkopen.2023.25158 PMC10366702

[104] Malik VS Popkin BM Bray GA Després JP Willett WC Hu FB. Sugar-sweetened beverages and risk of metabolic syndrome and type 2 diabetes: a meta-analysis. Diabetes Care 2010;33(11):2477–83. 20693348 10.2337/dc10-1079 PMC2963518

[105] Qin P Li Q Zhao Y Chen Q Sun X Liu Y Sugar and artificially sweetened beverages and risk of obesity, type 2 diabetes mellitus, hypertension, and all-cause mortality: a dose-response meta-analysis of prospective cohort studies. Eur J Epidemiol 2020;35(7):655–71. 32529512 10.1007/s10654-020-00655-y

[106] Hu Y Costenbader KH Gao X Al-Daabil M Sparks JA Solomon DH Sugar-sweetened soda consumption and risk of developing rheumatoid arthritis in women. Am J Clin Nutr 2014;100(3):959–67. 25030783 10.3945/ajcn.114.086918 PMC4135503

[107] Dey M Cutolo M Nikiphorou E. Beverages in rheumatoid arthritis: What to prefer or to avoid. Nutrients 2020;12(10):3155. 33076469 10.3390/nu12103155 PMC7602656

[108] Gómez M Gutkoski LC Bravo-Núñez Á. Understanding whole-wheat flour and its effect in breads: A review. Compr Rev Food Sci Food Saf 2020;19(6):3241–65. 33337058 10.1111/1541-4337.12625

[109] Bärebring L Winkvist A Gjertsson I Lindqvist HM. Poor dietary quality is associated with increased inflammation in Swedish patients with rheumatoid arthritis. Nutrients 2018;10(10):1535. 30340316 10.3390/nu10101535 PMC6213361

[110] Bustamante MF Agustín-Perez M Cedola F Coras R Narasimhan R Golshan S Design of an anti-inflammatory diet (ITIS diet) for patients with rheumatoid arthritis. Contemp Clin Trials Commun 2020; 17:100524. 32025586 10.1016/j.conctc.2020.100524 PMC6997513

[111] Aune D Keum N Giovannucci E Fadnes LT Boffetta P Greenwood DC Whole grain consumption and risk of cardiovascular disease, cancer, and all cause and cause specific mortality: Systematic review and dose-response meta-analysis of prospective studies. BMJ 2016;353:i2716. 27301975 10.1136/bmj.i2716 PMC4908315

[112] Santos CSP Cruz R Cunha SC Casal S. Effect of cooking on olive oil quality attributes. Food Res Int 2013;54(2):2013.

[113] Bahadori S Salamzadeh J Kamalinejad M Shams Ardekani MR Keshavarz M Ahmadzadeh A. Study of the effect of an oral formulation of fig and olive on rheumatoid arthritis (RA) remission indicators: A randomized clinical trial. Iran J Pharm Res 2016;15(3):537–45. PMC5149042 27980590

[114] De Vito R Fiori F Ferraroni M Cavalli S Caporali R Ingegnoli F Olive oil and nuts in rheumatoid arthritis disease activity. Nutrients 2023;15(4):963. 36839323 10.3390/nu15040963 PMC9962234

[115] Deoula MS El Kinany K Huybrechts I Gunter MJ Hatime Z Boudouaya HA Consumption of meat, traditional and modern processed meat and colorectal cancer risk among the Moroccan population: A large-scale case-control study. Int J Cancer 2020;146(5):1333–45. 31525258 10.1002/ijc.32689

[116] Pan L Chen L Lv J Pang Y Guo Y Pei P Association of red meat consumption, metabolic markers, and risk of cardiovascular diseases. Front Nutr 2022;9:833271. 35495958 10.3389/fnut.2022.833271 PMC9051033

[117] Ren GY Chen CY Chen GC Chen WG Pan A Pan CW Effect of flaxseed intervention on inflammatory marker C-reactive protein: A systematic review and meta-analysis of randomized controlled trials. Nutrients 2016;8(3):136. 26959052 10.3390/nu8030136 PMC4808865

[118] Nordström DCE Friman C Konttinen YT Alpha-linolenic acid in the treatment of rheumatoid arthritis: A double-blind, placebo-controlled and randomized study: flaxseed vs. safflower seed. Rheumatol Int 1995;14:231–4. 7597378 10.1007/BF00262088

[119] Ghaseminasab-Parizi M Nazarinia MA Akhlaghi M. The effect of flaxseed with or without anti-inflammatory diet in patients with rheumatoid arthritis: A randomized controlled trial. Eur J Nutr 2022; 61:1377–89. 34837524 10.1007/s00394-021-02707-9

[120] Askarpour M Karimi M Hadi A Ghaedi E Symonds ME Miraghajani M Effect of flaxseed supplementation on markers of inflammation and endothelial function: A systematic review and meta-analysis. Cytokine 2020;126:154922. 31739218 10.1016/j.cyto.2019.154922

[121] Zielińska M Dereń K Polak-Szczybyło E Stępień AE. The role of bioactive compounds of Nigella sativa in rheumatoid arthritis therapy: Current reports. Nutrients 2021;13(10):3369. 34684370 10.3390/nu13103369 PMC8539759

[122] Hadi V Kheirouri S Alizadeh M Khabbazi A Hosseini H. Effects of Nigella sativa oil extract on inflammatory cytokine response and oxidative stress status in patients with rheumatoid arthritis: A randomized, double-blind, placebo-controlled clinical trial. Avicenna J Phytomed 2016;6:34–43. 27247920 PMC4884216

[123] Kheirouri S Hadi V Alizadeh M. Immunomodulatory effect of Nigella sativa oil on T lymphocytes in patients with rheumatoid arthritis. Immunol Investig 2016;45:271–83. 27100726 10.3109/08820139.2016.1153649

[124] Mahdavi R Namazi N Alizadeh M Farajnia S. Nigella sativa oil with a calorie-restricted diet can improve biomarkers of systemic inflammation in obese women: A randomized double-blind, placebo-controlled clinical trial. J Clin Lipidol 2016;10(5):1203–11. 27678438 10.1016/j.jacl.2015.11.019

[125] Wirth MD Burch JB. Inflammatory potential of the diet: role of circadian rhythms and sleep. In: Hébert JR Hofseth LJ, editors. Diet, Inflammation, and Health. Academic Press; 2022. p. 747–85. doi:10.1016/B978-0-12-822130-3.00013-2.

[126] Baxter M Ray DW. Circadian rhythms in innate immunity and stress responses. Immunology 2020;161(4):261–7. doi:10.1111/imm.13166. 31820826 PMC7692257

